# Single administration vaccines: delivery challenges, in vivo performance, and translational considerations

**DOI:** 10.1080/14760584.2023.2229431

**Published:** 2023-07-04

**Authors:** Kyprianos Michaelides, Maruthi Prasanna, Raj Badhan, Afzal-Ur-Rahman Mohammed, Adam Walters, M. Keith Howard, Pawan Dulal, Ali Al-Khattawi

**Affiliations:** a School of Pharmacy, Aston University, Birmingham, UK; b AVaxziPen Limited, Milton Park, Abingdon, Oxfordshire, UK; c AstraZeneca, Aaron Klug Building, Cambridge, UK

**Keywords:** Single administration vaccines, vaccine controlled release, vaccine stability, commercialization, Hepatitis B vaccine, multiple dose vaccine, microsphere formulation, PLGA

## Abstract

**Introduction:**

With a limited global supply of vaccines and an increasing vaccine hesitancy, improving vaccination coverage has become a priority. Current vaccination regimes require multiple doses to be administered in a defined schedule where missed doses may lead to incomplete vaccine coverage and failure of immunization programmes. As such, there is an ever-increasing demand to convert multi-dose injectable vaccines into single-dose formats, often called single administration vaccines (SAVs).

**Areas covered:**

This review summarizes recent developments in the field of SAVs, with a focus on pulsatile or controlled-release formulations. It will identify the technical challenges, translational as well as commercial barriers to SAVs development. Furthermore, the progress of SAV formulations for hepatitis B and polio vaccines will be reviewed thoroughly as case studies, with a focus on the development challenges and the preclinical immunogenicity/reactogenicity data.

**Expert opinion:**

Despite the efforts to develop SAVs, few attempts have advanced to Phase-I trials. Considering the SAV development journey and bottlenecks, including commercial barriers from the early stages, may overcome some of the hurdles around the technology. The renewed global focus on vaccines since the COVID-19 pandemic could facilitate development of a new generation of technologies for pandemic preparedness including strategies for SAVs.

## 1. Introduction

Many lifesaving vaccines such as those for Hepatitis B and Polio require administration of multiple doses over a period ranging from weeks to months. This creates both logistical and public health challenges for health authorities in communities where there is lack of sufficient primary health infrastructure. The issues are exacerbated during public health emergencies such as pandemics, natural disasters, or political unrest. These issues could be mitigated by development of single administration vaccines (SAVs). The primary aim of the SAV approach is to decrease the number of booster doses required, resulting in better compliance to the vaccination schedule and reduced logistics associated with multiple administration of doses [1].

For better appreciation of the impact multiple vaccine doses has on immunity a brief overview of the relevant immune system mechanisms is discussed below. Normally, the protection against infectious organisms is mediated through two mechanisms: the innate system, which provides a general, rapid initial defense against potentially pathogenic microorganisms, and the adaptive immune system, which is a slower but highly specific response to antigens and generates long-term memory against the same antigen. The generation of a successful immune response against a pathogen involves a close cooperation between the two systems with the participation of different immune cells, chemokines, cytokines, and chemical mediators.

In principle, when the immune system encounters an antigen in the form of a pathogen or a vaccine, different arms of the innate immune system tackle it depending on the type of antigen or the route of entry. In the chain of activation, naïve B-cells sense the antigen using their relevant receptors and differentiate into antibody producing plasma cells and memory B-cells with the help of T-cells. The antibodies produced at the early stage are predominantly immunoglobulin IgM with low binding affinity which can switch toward more specific immunoglobulin IgG based on the persistence of the antigen. In the absence of antigen, this primary immune response subsides leading to decline in the levels of antibodies due to apoptosis of antibody producing B cells.

Upon subsequent exposure to the same antigen, antigen-specific memory B-cells rapidly proliferate, resulting in the production of abundant and highly specific IgG antibodies. This secondary immune response also generates a larger pool of memory B-cells specifically targeting the antigen for future encounters [2]. This highlights the importance of completing all vaccine doses in order to stimulate a robust memory response and achieve optimal effectiveness in protecting against a specific disease.

**Figure 1.**
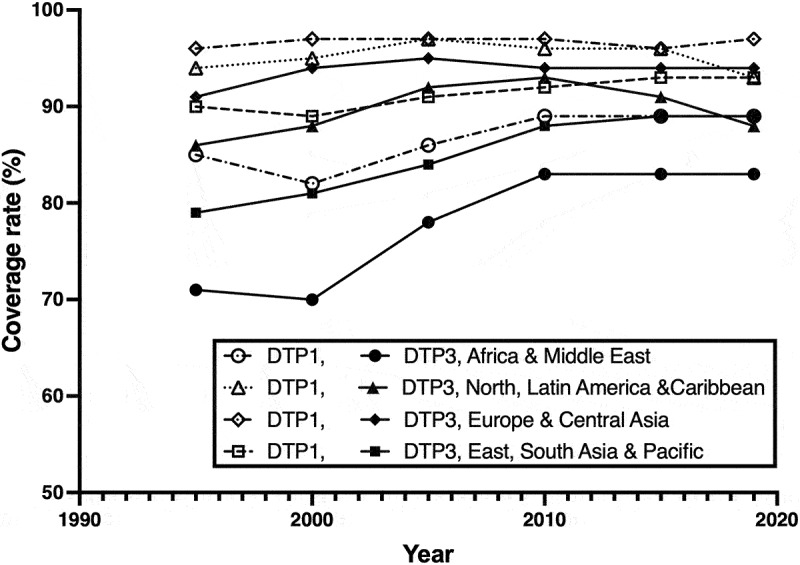
Coverage rates of DTP vaccine at 5-year intervals in different world regions (1995–2019) generated from data extracted from UNICEF database. DTP1, Diphtheria/Tetanus/Pertussis dose 1; DTP3, Diphtheria/Tetanus/Pertussis dose 3.Note: Data obtained from https://data.unicef.org/resources/dataset/immunization/

As illustrated in Figure 1, using data extracted from UNICEF’s database, the coverage for the first dose of Diphtheria/Tetanus/Pertussis vaccine (DTP1) is 5–10% higher than that for the final dose (DTP3) globally between 2015 and 2019 regardless of the geographic location. This can have major consequences, especially for diseases like diphtheria with a mortality rate up to 20% [3]. In 1980s, the World Health Organisation (WHO) identified the importance of such approaches and introduced the formulation of single-dose vaccines as a goal for its Special Programme for Vaccine Development [1]. Unfortunately, despite considerable efforts from various research groups, the successful commercialization and implementation of the SAV technology in clinical settings has not yet been achieved. Recently, the biopharmaceutical industry has made huge advancements in the field of vaccine manufacturing, particularly during the COVID-19 pandemic. As such there has been a renewed interest in the development of SAV.

To date the most tested SAV technologies have been based on biodegradable microspheres or nanospheres that prolong the release of antigens over an extended period or release antigen in discrete pulses. Candidate systems include liposome, lipid nano particles, and polymer-based particles; nonparticulate systems include microneedles and *in situ* gelling system [2–5]. Among these technologies, poly lactic-co-glycolic acid (PLGA)-based strategy has shown the most promise and is the most widely investigated [4–7].

However, despite promising preclinical data from both academia and industry, there is no marketed SAV product [8]. Many groups have reviewed issues underpinning the failure of SAVs to materialize into a commercial product. The issues reviewed vary from specific challenges with biodegradable polymers e.g. the acidic environment created by the degradation of PLGA combined with the instability of antigens, to manufacturing and cost constraints [9–11]. However, there has been insufficient discussion regarding the challenges associated with formulation development, the attainment of the desired immune profile, and the translational/commercial aspects crucial for the success of SAV technology as a viable clinical solution.

The current review aims to discuss the major challenges in the formulation of SAV, and different strategies adopted by research groups with special emphasis on PLGA based approaches. Subsequently, *in vivo* responses to SAV technologies are discussed focusing on immunogenicity and reactogenicity. The current potential candidates for SAV technologies are parenterally administered vaccines that require more than one dose within a period of 6 months. A summary of these candidates and their recommended schedules is highlighted in Table 1. This includes seven diseases covered in routine immunization schedules in developed countries, such as hepatitis B, diphtheria, tetanus, whooping cough, Haemophilus influenza type b, polio, human papillomavirus and four conditional vaccines such as varicella zoster, Japanese encephalitis, rabies, and dengue vaccines.

**Table 1.** List of potential vaccine candidates for SAV formulation.

	Vaccine		Type	Recommended schedule
Routine immunization	Hepatitis B vaccine		Recombinant Subunit	Primary series of 3 doses: - As soon as possible after birth - Gap of 4 weeks between doses Unvaccinated individuals vaccinated with a 0, 1, 6-month schedule
	Diphtheria (DTP)		Toxoid	Primary series of 3 doses: - Earliest start: 6 weeks of age - Gap of 4 weeks between doses
	Tetanus (DTP)			
	Whooping cough (DTP)			
	Haemophilus influenza type b (Hib)		Subunit/conjugate	Primary series of 3 dose - Earliest start: 6 weeks of age - Gap of 4 weeks between doses
	Polio (IPV)		Inactivated	Primary series of 3 doses: - Earliest start: 8 weeks of age - Gap of 4 weeks between doses Course of 3 doses given to unimmunized adults
	Human Papillomavirus (HPV)		Recombinant Subunit	Prime dose given at the age 12–13 followed by a booster dose after at least 6 months. A three-dose schedule (0, 1–2, 6 months) used for vaccinations initiated ≥15 years of age or in immunocompromised people.
Conditional*	Varicella Zoster vaccine		Live Attenuated	Second dose 4 to 8 weeks after the prime
	Japanese encephalitis vaccine		Inactivated	Second dose 28 days after the prime, but people aged 18–64 can have the second dose 7 days after prime.
	Rabies vaccine		Inactivated	Three doses over a period of 28 days
	Dengue vaccine (CYD-TDV)		Live attenuated	Prime dose after 9 years of age followed by a second dose 6 months later (plus booster dose at 12 months)
Pandemic	COVID-19 vaccine	Spikevax (Moderna)	mRNA	Second dose 28 days after the prime.
		Comirnaty (Pfizer/BioNTech)	mRNA	Second dose at least 21 days after the prime
		Vaxzevria (Oxford/AstraZeneca)	Adenovirus-vectored DNA	Second dose 4 to 12 weeks after the prime
		Jcovden (Janssen)	Adenovirus-vectored DNA	Booster dose at least 2 months after the prime
		Nuvaxovid (Novavax)	Recombinant Subunit	Second dose 3 weeks after the prime
		COVID-19 Vaccine Valneva (Valneva)	Inactivated	Second dose 4 weeks after the prime

Note: *Conditional depending on risk factors e.g. location, exposure.

During the COVID-19 pandemic, it has been observed that the administration of multiple booster doses of the initial vaccine generations has been effective in reducing hospitalization and improving multiple patient outcomes, even in the presence of emerging variants [12–14]. In addition, sustained delivery of subunit receptor-binding domain (RBD) of the SARS-CoV-2 spike protein has been shown to elicit potent anti-RBD and anti-spike antibody titers; providing broader protection against SARS-CoV-2 variants compared to conventional bolus administration of the same vaccine [15]. Moreover, the recent development of COVID-19 vaccines illustrates how the number of vaccines that would benefit from the implementation of SAV technology is likely to increase.

As shown in Table 1, each vaccine has its own distinctive characteristics and administration schedule, which implies that any SAV technology would need to be customized to fulfil the specific chemical, physical, and immunogenic requirements of the vaccine. One particular requirement that arises is the inclusion of adjuvants in nearly all subunit vaccines, as they are essential for generating a robust primary and secondary immune response, thereby enhancing protection and longevity of the immune response.

Two case studies will then be reviewed in greater detail, a subunit, and an inactivated vaccine for the prevention of polio and hepatitis b, respectively. To conclude, a summary of the challenges that must be addressed before an SAV formulation could transition into a commercial product has been included.

## 2. Formulation challenges of SAV technologies

Adapting a conventional vaccine to an SAV requires the design of a controlled-release system that can maintain antigen stability throughout the process. Both release profile and stability are discussed in the following sections as key features for generating a strong and long-lasting immune response.

### 2.1. Controlling release of antigens to meet the demands of a multiple dose vaccine

A range of controlled release technologies have been investigated, consisting mainly of natural/synthetic polymers and lipid carriers. However, only a limited number of these polymers or carriers are included in United States Food and Drug Administration (FDA) generally recognized as safe list. Natural materials such as chitosan and alginates have been used by some groups, particularly because they are cheaper and more readily available than synthetic polymers. Evidence of sustained release from chitosan microspheres for 6 months was reported for *in vitro* and *in vivo* studies with tetanus toxoid vaccine [16]. However, the batch-to-batch variability of these natural polymers and the difficulty in tuning their release mechanism limits their potential as commercial SAV technologies [17]. On the other hand, synthetic biodegradable materials such as polycaprolactone (PCL) and polyorthoesters can be tuned to optimize release kinetics. PCL is a biodegradable polyester frequently used in medical devices for tissue engineering such as sutures [18]. Bansal et al. explored PCL in SAV technology for a tetanus vaccine due to its slow-release properties and ability to produce degradation products that are less acidic in comparison to other biodegradable polymers. Both *in vivo* and *in vitro* studies provided promising results by showing sustained release over 30 days and improving both immune response and survival rates in mice models [19]. Tomar et al. achieved *in vitro* release of Hepatitis B surface antigen (HBsAg) for a period of 6 months and elicited an immune response comparable to the conventional HBsAg aluminum vaccine *in vivo* [20]. However, PCL use in vaccine delivery is limited to a handful of studies and its use has been surpassed by PLGA.

PLGA is the most commonly used polymer in long-acting injections because of its safety profile, biocompatibility, and biodegradability. It has been successfully used in commercial products for non-vaccine-controlled release applications such as Lupron®, Trelstar®, Bydureon®, Signifor® LAR, Sandostatin® LAR, Somatuline® Depot for more than 30 years [21]. Furthermore, PLGA is a synthetic polymer allowing tuning of the release profile by changing its characteristics such as the molecular weight, lactic/glycolic acid ratio or polymer end chemistries [22].

#### 2.1.1. PLGA release kinetics for use in SAV

**Figure 2.**
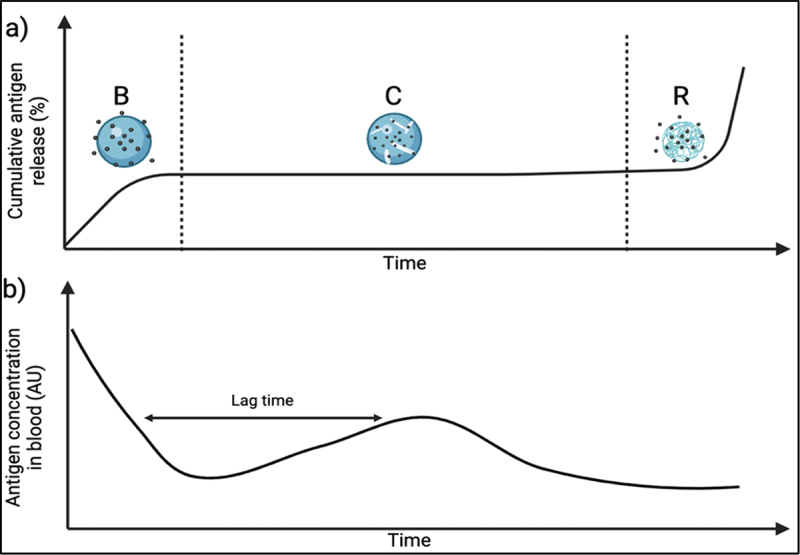
(a) Theoretical Triphasic release pattern from PLGA particles: initial antigen release from the surface of the microparticles through diffusion resulting in a burst phase (b), diffusion-dependent constant release phase (c), second rapid release phase caused by polymer erosion (R); (b) Corresponding theoretical antigen concentration in blood.

There are two main release modes for a hydrophilic antigen from PLGA microspheres. Firstly, diffusion, which is the transport of antigen through aqueous channels (formed upon hydrolysis) in the polymer [23]. Secondly, erosion, which involves the molecular breakdown of PLGA matrices. Those release modes impact the release profile and lead to a triphasic release pattern (Figure 2a). This includes an initial burst that is a result of the rapid diffusion of antigen from the surface of PLGA microspheres and depends mainly on antigen surface enrichment (Figure 2a, B). This is followed by a constant release phase of the antigen that is again governed by diffusion (Figure 2a, C). This phase lasts until polymer erosion begins, signaling the initiation of the second rapid phase (Figure 2a, R) [24,25]. In most sustained release applications an excessive initial burst release is considered undesirable due to potential toxicity and efficacy implications [26,27]. The release profile from PLGA microspheres translates to an *in vivo* antigen concentration profile (Figure 2b).

The factors governing the release from PLGA include its physicochemical properties or those of the antigen, and the environment to which the release system is exposed. In terms of delivery system attributes, the size of microspheres affects the three different release phases. A smaller particle size and higher associated surface area often leads to a bigger initial burst followed by rapid diffusion-dependent release. In contrast, larger microspheres usually display a sigmoidal release profile that is governed by both diffusion and erosion [28]. Experiments conducted by Uchida et al. showed that burst release of a model antigen (ovalbumin) decreased from 80% to 20% when increasing the average particle size from 2.1 to 14.3 μm [29]. Erosion involves the degradation of ester bonds between lactic and glycolic acids by hydrolysis and the formation of free acids. The acids act as hydrolytic catalysts themselves and can lead to heterogenous degradation (more degradation in the center due to the acidic environment created than at the surface of the microspheres, where the pH is closer to neutral i.e. bulk erosion), which is most often observed in microspheres of larger sizes [30,31]. Antigen loading is another release modulator primarily affecting the burst release phase. At low antigen loading, a diminished initial burst release has been observed attributed to lower surface enrichment. On the other hand, a larger initial burst and increased release rate have been detected at higher antigen loading [32]. Guarecuco et al. observed that by increasing percentage loading of a model antigen (bovine serum albumin) from 0.5 to 5% w/w, the initial burst release percentage increased from 28.0% to 63.7% [33]. The distribution of the antigen can also play a major role in the release profile: antigen surface enrichment of the PLGA causes high initial burst, whereas formation of a core-shell structure delays release [34]. Furthermore, the polymer density is a modulator of release profiles because porosity directly affects the diffusion of hydrophilic antigens. The lower the density and higher porosity, the higher the release by diffusion. Pore formation is caused by PLGA degradation and water absorption. Some reports mention another phenomenon of pore closure possibly happening due to the generation of a less porous surface layer attributed to the non-homogenous degradation or due to the structural collapse and mobility of the polymer [35].

**Figure 3.**
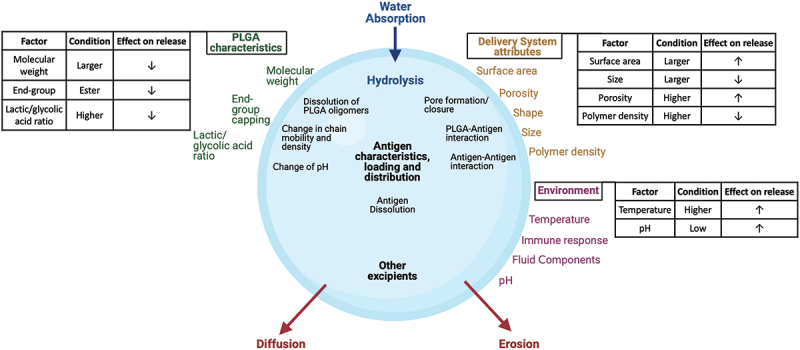
Factors influencing antigen release from PLGA microspheres.

There is a variety of other factors that contribute to the release profile (Figure 3): starting from the polymer characteristics, such as lactic/glycolic acid ratio, ending groups, and molecular weight. Higher lactic/glycolic ratio and molecular weight, as well as capped ending groups, lead to a more hydrophobic PLGA with less water absorption capacity. Consequently, hydrolysis and erosion of the polymer backbone is reduced [36]. Most studies for SAV formulations and marketed long acting injectables focus on or use ratios from 50:50 to 100:0 (Polylactide, PLA), molecular weights under 100 kDA and free carboxylic acid- or ester-capped end groups to generate a sustained/pulsatile release profile of 6 months or less. The molecular weight, hydrophilicity, and the ability of the antigen to interact (H-bonding, Vander-Waals, and hydrophobic interactions) with PLGA can also determine diffusion rates [37,38]. Other excipients included in the formulation, such as stabilizers, can have a release modulating role as well [24]. Jaganathan et al. observed that increasing trehalose concentration to 2% resulted in high burst release (80 ± 2.8%) of Hepatitis B antigen [39]. This could be due to the hydrophilicity of the added trehalose and its potential to act as a porogen on the PLGA microspheres, facilitating both diffusion and erosion [40].

The physiological environment around PLGA microspheres *in vivo*, such as temperature and pH, also impact the release profile. This may explain observed differences between *in vitro* and *in vivo* release. Usually, *in vivo* release is faster owing to a number of factors that accelerate the degradation of biodegradable PLGA, such as reactive oxygen species produced by macrophages and neutrophils involved in the immune response [41]. The presence of enzymes such as lipases and esterases in low levels may also exhibit biodegradative effects on polymers [42,43]. Therefore, a robust technology tackling most of the aforementioned challenges is required to achieve precise release control *in vivo*. This is not trivial given the lack of appropriate *in vitro-in vivo* correlations for long-acting and pulsatile release injectable systems [44,45].

Vaccination regimen for all currently licensed vaccines include the first dose to prime the immune system followed by subsequent booster doses after a suitable interval. While for most small molecule active pharmaceutical ingredients a zero-order release is optimum, for vaccines a triphasic release profile or a discrete pulse release profile is considered more desirable. However, sustained release of antigen over a period of time has been shown to have better impact on immunogenicity and is increasingly receiving attention [46,47].

### 2.2. Maintaining physical and chemical stability of antigens

Many of the antigens currently used in vaccination are proteins [48]. A successful formulation of such vaccine entities must maintain physical and chemical stability of the antigen during the stages of preparation, storage and *in vivo* environment after administration. There are two main processes that could lead to the loss of antigen immunogenicity. The first is denaturation, which involves the alteration of secondary, tertiary, or quaternary structure, and degradation, which involves covalent modification. Denaturation could be the outcome of aggregation, unfolding, precipitation and surface adsorption [49], whereas degradation is the result of reactions such as acylation, deamidation, oxidation, and hydrolysis of amide bonds [50].

During SAV preparation, the main causes of antigen instability are the processing temperature, shear forces applied, and usage of organic solvents. Each antigen has a distinct stability profile, but generally avoiding extreme temperatures, using lower shear forces and limited use of organic solvents could reduce the chances of protein unfolding and aggregation [11]. Maintaining antigen stability during storage is another noteworthy challenge for SAV technologies containing excipients that may catalyze the degradation of proteins. Moisture and temperature elevation may catalyze hydrolysis of the PLGA polymer. This can lead to conditions inside the polymer matrix that are usually anticipated during the active release phase and can affect protein stability. This may involve a drop in pH which can lead to the chemical degradation of proteins via hydrolysis and aggregation [51]. At low pH, the interaction between PLGA and protein may be favored, leading to the formation of amide linkages between the two. The pH drop is coupled with increased temperature and humidity, escalating the chance of protein destabilization *in vivo* [52]. To counter the pH drop associated with PLGA hydrolysis, buffering agents have been introduced. Excipients such as magnesium hydroxide have shown to prevent conformational changes, deamidation, and aggregation of antigens [1,53]. To overcome protein stability issues, a variety of stabilizers have been introduced.

Both sugars and surfactants have been used to protect proteins when operating at extreme temperatures and drying conditions during preparation. Non-ionic surfactants such as polysorbate 20/80 can reduce interface-induced protein aggregation, whereas sugars (e.g. trehalose, inulin, sucrose) can have a dual role. When sugars are at the glassy state (below glass transition temperature) they form an amorphous sugar glass matrix around the protein, restricting its mobility [54]. Another role of sugars that enables them to preserve protein integrity is the formation of hydrogen bonds between the hydroxyl groups of a stabilizing sugar and the polar groups of the protein, enabling the protein to maintain its native structure [55,56]. This phenomenon is often referred to as the water replacement hypothesis and is particularly important in preventing destabilization of proteins by water loss during processes such as drying. In most of the previous SAV attempts trehalose dominated the area of stabilizing excipients in PLGA formulations with concentrations of 20% w/w or less. Careful consideration has to be taken in selecting the type and concentration of the stabilizers as they may also have an impact on the release mechanism (e.g. trehalose is hygroscopic).

Over the last few decades several methods have been employed by formulation scientists to produce SAV formulations with the desired attributes of antigen stability and pulsatile/continuous release profile. There is a handful of notable SAV approaches that attempted to address both key features, release, and stability, generating a strong and long-lasting immune response in small animal studies (Table 2). In 2000, a group created tetanus toxoid SAV formulations with evidence of sustained release for 12 weeks. The most effective formulations were microspheres co-adjuvanted with alum and a size of less than 5 μm prepared using PLGA 50:50. Despite generating antibody titers higher than the protection limit, there is no evidence of the approach proceeding to clinical trials due to economic and technological reasons [10,57]. A more recent approach employing double emulsion managed to achieve pulsatile release profiles for inactivated polio vaccine (IPV) both *in vitro* and *in vivo*. Cationic polymer excipients such as Eudragit E, poly (L-lysine), and branched poly-ethylenimine (bPEI) were used to modulate release from PLGA microspheres and prevent protein aggregation caused by low pH. Eudragit E acts as a base due to its amine functional groups causing an increase in the pH which can accelerate PLGA degradation by base catalyzed hydrolysis. Also, Eudragit E dissolution generates channels for IPV to escape from the acidic environment in the microsphere by diffusion [58,59]. Others have employed alternative formulation methods, such as StampEd Assembly of polymer Layers to produce core-shell microparticles capable of releasing antigens at desired timepoints within 2 months. This approach also reported higher stability of the antigen compared to emulsification processes due to the elimination of organic solvents and other emulsification stressors [60]. The atomic layer deposition (ALD) method employed by another group used alumina layers instead of a biodegradable polymer as a release technology, showing similar or better immune response to the conventional vaccine. Coating antigens with alumina can also provide adjuvant effects as well as increasing stability of antigens because the coating is impermeable to water vapor [61]. The aforementioned approaches and dozens of other SAV technologies pursue a similar vaccine development path. The formulation stage is followed by *in vitro* and *in vivo* testing to assess immunogenicity. Eliciting a strong and long-lasting immune response is fundamental for any vaccine candidate and is the key to being able to proceed to the next stage and face another check point: the translational aspects and commercialization. The cost effectiveness and scalability of some of the above methods, especially the ones employing novel/non-conventional manufacturing processes, may place considerable challenges for translating them into a commercial SAV technology.

**Table 2.** Notable Single administration vaccine approaches.

Antigen	Excipients		Microparticle fabrication method	Release profile	Immune response	Pros (+)/Cons (−)	Reference
	Stabilizers	Release technology					
Tetanus toxoid	Trehalose, albumin, poloxamer 188, stabilizer no. 1 (commercial mixture of saccharides, amino acids, and electrolytes), stabilizer no. 176 (commercial mixture of saccharides, amino acids, electrolytes, and a protein)	PLGA 50:50, PLA	Spray-drying, Solvent evaporation, Coacervation	N/A	-All formulations generated antibody titers higher than the protection limit maintained for 12 weeks. -Tetanus toxin neutralization capacity Control -> 20 IU/ml SAV -> 5–15 IU/ml	(+) Various combinations of stabilizers, release technologies, and microparticle fabrication methods were compared	[57]
IPV serotypes 1,2,3	- Sucrose, sorbitol, trehalose, maltodextrin in combination with monosodium glutamate and magnesium Chloride were effective on maintaining stability of IPV on incubation at 37°C, sonication and vacuum drying. - Gelatin: did not show improvement in stability during the incubation but prevented loss during drying and sonication	PLGA 50:50 with Eudragit E	Double emulsion	EPO allowed tuning of release kinetics: − 7.5% EPO 2nd peak → 11 days − 5% EPO 2nd peak → 14–18 days − 3% EPO 2nd peak → 25 days Addition of arginine: − 2nd peak → 32 days.	The formulation: (8% maltodextrin, 6.8% MSG, 6.8% MgCl_2_, 3% EPO) displayed a non-inferior response to the control as measured by ELISA.	(+) Excipients tested in different combinations and concentrations. (−) Stabilizer’s screening (incubation at 37°C, sonication and drying process) to evaluate excipients stabilizing performance not done in the presence of PLGA	[58]
IPV serotypes 1,2,3	- Cationic polymer excipients Eudragit E, poly (L-lysine), and branched poly-ethylenimine were used to increase stability and prevent protein aggregation caused by low pH. - Small-molecule excipients for IPV protection (17% MSG, 20% maltodextrin, and 17% MgCl_2_)	PLGA 50:50 with EPO or PLL + bPEI	Double emulsion	Pulsatile release was observed as the three IPV serotypes were released in two distinct bursts. − 2nd peak → 20–30 days	Non-inferior immune response to the control for both SAV formulations.	(+) Use of cationic polymers to modulate and achieve pulsatile release	[59]
IPV and Ovalbumin	Sucrose, monosodium glutamate, and magnesium chloride.	PLGA: − 50:50 ester end group − 50:50 acid end group − 85:15 acid end group	StampEd Assembly of polymer Layers (SEAL)	Fluorescently labeled dextran: *In vitro*: no burst release, day 10 (1st peak), day 15 (2nd peak) day 34 (3rd peak) *In vivo*: no burst release, day 9 (1st peak), day 20 (2nd peak) day 41 (3rd peak)	Peak titers were higher than the control (two-dose boluses)	(+) Pulsatile release: delayed bursts without prior leakage (i.e. delayed sharp bursts on a specific day) (−) Non-traditional manufacturing process platform likely to be related with cost-effectiveness and scalability	[60]
HPV16 L1 Capsomere (model antigen)	-Trehalose (protect antigen during drying) -Hydroxyethyl starch (raises glass transition temperature increasing stability and preventing agglomeration)	-Trimethyl-aluminium	- Spray-drying (to stabilize the antigen) - Atomic layer deposition	HPV16 L1 labeled with IR Dye 800CW -*In vivo*: No coats ≈ 1–3 weeks 100 coats ≈ 4 weeks 250–500 coats ≈ 14 weeks	-Antibody titers similar/better to the control (prime + booster dose)	(+) Alumina layers acted both as adjuvants and as release technology (−) Uncontrolled fracture of particles in lab-scale atomic layer deposition reactor	[61]

Note: IPV, inactivated polio vaccine; SAV, single administration vaccine; EPO, Eudragit E; MSG, monosodium glutamate; MgCl_2_, magnesium chloride; ELISA, enzyme-linked immunosorbent assay; PLL, poly (L-lysine); bPEI, poly-ethylenimine; PLGA, Poly lactic glycolic acid; PLA, poly lactic acid.

## 3. In vivo responses to SAV technologies

### 3.1. Immunogenicity in small animal studies

Formulations that provide promising results *in vitro* could be moved on to animal testing to assess their immunogenicity and reactogenicity. Animal models may provide an idea of the cellular responses expected in humans. Unfortunately, no SAV technology has ever progressed beyond preclinical testing due to failing to meet the desired immune response or overcome technological and economic barriers [1,10]. To illustrate the challenges expected during immunogenicity studies, two examples of vaccine candidates suitable for SAV formulations are being discussed in the next two sub-sections.

#### 3.1.1. Hepatitis B (HBV): A stable target for SAV technologies

HBV immunization uses a virus-like-particle (VLP) composed of surface protein from hepatitis B virus. The vaccination regime requires a prime followed by two booster doses to provide sufficient protection. The ideal HBV immunization is expected to generate sufficient antibodies as quickly as possible that persist, and offer equivalent protection to naturally acquired antibodies [62]. The correlate of protection in vaccinated humans is an antibody response greater than 10 IU/L [63]. HBV immunization is implemented in almost all the national vaccination schedules around the world and is highly effective (95%) in providing protection from chronic hepatitis B infection for at least two decades [64]. According to the WHO, the global coverage with three doses of hepatitis B vaccine was estimated to be 85% in 2019 [65]. Although this is a great achievement for public health, it is important to note that millions of new-borns remain unprotected every year [66]. A contributor to this issue is the incomplete immunization due to failure to vaccinate with subsequent doses. One successful and implemented approach is the use of combination vaccines (containing antigens for two or more diseases) to improve coverage rates [67]. An example of a combination vaccine is the 6-in-1 vaccination providing protection for hepatitis B, polio, diphtheria, tetanus, pertussis, and haemophilus influenzae type b after a course of three bolus injections (for babies at 8, 12, and 16 weeks old). Due to its suitable vaccination regimen HBsAg has been a common model for investigation of SAV formulations. Furthermore, the HBV VLP is relative stability in acidic environment lending itself to formulation in PLGA.

For example, Feng et al. developed SAV formulations for the continuous release of HBsAg subunit vaccine and tested their immunogenicity in mice (via subcutaneous route). The three candidate SAV formulations were developed using a double emulsion technique for microencapsulation of the antigen in PLGA microspheres. Two of the formulations had the same lactic: glycolic ratio (50:50) but different polymer end groups (carboxylic acid and ester capped end groups). The third formulation was made of PLGA (75:25) and ester capped end groups. A mixture of the three formulations managed to produce similar mean antibody titers to the active control which received three bolus injections (2.5 μg of the HBsAg aluminum-vaccine) at 0, 1, and 2 months. The immune responses generated were on par with the active control for the 4-month duration of the experiment [68].

Using a similar approach, it was demonstrated that an SAV formulation of HBsAg antigens in microspheres can maintain an immune response at levels analogous to the conventional three-bolus intramuscular injection with alum (0, 1, 6 months) for at least 12 months after a single injection. To qualitatively measure the antibodies generated from SAV, Singh et al. developed an inhibition assay to show that the antibody binding specificity to HBsAg of both the SAV and the control were comparable. The antibodies produced by mice after exposure to SAV were also investigated using competitive ELISA. The aim of this assay was to determine whether the antibodies induced were able to compete with murine antibodies specific to HBsAg neutralizing region. The rationale for this was that if both compete for the same epitope, then the antibodies generated from SAV can provide protection to the actual virus. The results revealed that both the neutralizing monoclonal antibodies and the sera from SAV-vaccinated animals displayed similar specificity [69].

The inclusion of stabilizing excipients has also been trialed in the HBV model; for example Jaganathan et al. included trehalose and magnesium hydroxide and evaluated immunogenicity in guinea pigs by comparing it to the active control of two doses of the conventional vaccine. The results revealed that over the course of the 90-day experiment, both the control and the SAV showed an equivalent, significant elevation of anti-HBsAg antibodies [39].

A more recent study by Zheng et al. developed an SAV formulation capable of eliciting a more comprehensive and long-lasting immune response compared to two doses of the conventional vaccine with a 4-week interval. The immunogenicity results of the control and the SAV formulation were comparable throughout the 90 days of the experiment, but IgG2a levels induced by SAV gradually increased, while those of the control group decreased over time [70].

It is known that the size of the particle in the formulation can affect immunogenicity and clearance rate, to address this Kanchan and Panda compared a single injection of antigen-loaded nanospheres (200–600 nm) to antigen-loaded microspheres (2–8 μm). According to their observations, the majority of nanospheres were endocytosed by macrophages. This promoted a tendency toward cellular response, which was accompanied with Interferon-γ secretion. Consequently, IgG antibody titers were lower when compared to the microspheres that remained out of the cells or attached on the surface of antigen presenting cells, slowly releasing their load encouraging a humoral response. Interestingly, neither of the unadjuvanted particulate formulations provided immunogenicity comparable to three doses of alum-adsorbed HBsAg. Only when the microspheres were mixed with alum was an immune response analogous to the control observed [71].

As noted above, many studies have generated promising data with HBV-PLGA formulations highlighting additional benefits of SAVs (Table 3). Indeed, some reports indicate that PLGA itself can act as an adjuvant in addition to its controlled release properties [73]. The potential mechanism of the immune enhancement by PLGA could be based on imitating the dimensions, shape, charge, and other characteristics of pathogens to increase cross presentation and uptake [74–76]. The prospect that SAV technologies can increase effectiveness via their adjuvant effect or continuous antigen exposure might be beneficial for populations that have high non-response rates to the conventional vaccine, such as smokers, the obese, and the elderly.

**Table 3.** Animal studies of hepatitis B single administration vaccines (SAVs).

SAV formulation	Immune response	Control	Duration	Model (route)	Ref.
HBsAg(7.5 μg) PLGA 50/50-COOH microspheres	Inferior results after the sixth week	2.5 μg HBsAg aluminium-vaccine at 0, 1, 2 months	4 months	Mouse (SC)	[68]
HBsAg(7.5 μg) PLGA 50/50 microspheres	Comparable serum antibody titres to control				
HBsAg(7.5 μg) PLGA 75/25 microspheres					
HBsAg(7.5 μg) mixture of PLGA 50/50, PLGA 75/25, PLGA 50/50 - COOH microspheres					
HBsAg(30 μg) PLG505, PLG858 (<10 μm) and PLA208 (>10 μm) microspheres	Antibody response comparable to control	10 μg HBsAg aluminium-vaccine at 0, 1, 6 months	12 months	Mouse (IM)	[69]
HBsAg(30 μg) PLG505 (<10 μm) and PLG858, PLA208 (>10 μm) microspheres					
HBsAg(20 μg) PLGA 50/50 with trehalose and Mg(OH)_2_ microspheres	Immune response comparable to the control	10 μg HBsAg aluminium-vaccine at 0 and 1 months	3 months	Guinea pig (SC)	[39]
HBsAg(20 μg) PLGA 50/50 with Mg(OH)_2_ only	Significantly lower immune response than the control				
HBsAg(20 μg) PLGA 50/50 with trehalose only					
HBsAg(10 μg) Alginate–chitosan–PLGA (50/50, 70/30) composite microspheres	Antibody levels comparable to control	5 μg HBsAg aluminium-vaccine at 0 and 1 months	3 months	Mouse (SC)	[70]
HBsAg(10μg) PLGA (50/50, 70/30) microspheres	Significantly lower at all sampling times				
HBsAg(20 μg) adsorbed microspheres: PLGA 50/50	Comparable immune response	10 μg HBsAg aluminium-vaccine at 0 and 1 months	42 days	Mouse (SC)	[72]
HBsAg(20 μg) adsorbed microspheres: PLGA 75/25					
HBsAg(1 μg) loaded PLA nanospheres	Inferior antibody titers	Total 1 μg HBsAg aluminium-vaccine at 0, 1, 6 months	6 months	Rat (IM)	[71]
HBsAg(1 μg) loaded PLA nanospheres with alum					
HBsAg(1 μg) loaded PLA microspheres					
HBsAg(1 μg) loaded PLA microspheres with alum	Comparable antibody titres to the control				

Note: SC, subcutaneous; IM, intramuscular; HBsAg, hepatitis surface antigen; PLGA, poly lactic glycolic acid; PLA, poly lactic acid.

In all the aforementioned studies, the HBV SAV formulations employ continuous release rather than a pulsatile release kinetic. The long-term immunological consequences of this format of delivery are still unknown in humans and there are potential concerns as to whether this type of dosing may induce tolerance. Early studies suggested that continuous exposure to large amounts of antigen in animals could result in building up of tolerance causing temporary immunological hyporesponsiveness or leading to immunological paralysis [77,78]. However, more recent studies have not reported these effects [79]. Addressing these questions may help to optimize SAV formulations and establish their efficacy and safety profile. In the current guidance from the WHO, it is optimal to have at least 4 weeks between the prime and booster doses to allow maturation of B memory cells generating higher secondary immune responses [80]. According to this paradigm, pulsatile SAV technologies may be favored over the continuous release approaches. Therefore, a pulsatile release technology mimicking multi-dose vaccine profiles appears to be preferable from a regulatory approval standpoint [1]. However, the need to further explore the association of the release profile with the immune profile is of fundamental importance.

#### 3.1.2. Inactivated polio vaccine (IPV): a key candidate for SAV technologies

Inactivated polio vaccine consists of three antigens to provide immunity for polio serotypes 1, 2, and 3. Each of these serotypes has a different stability profile, making the translation to a SAV technology more challenging. In the attempt to eradicate polio, the WHO tried to utilize both available vaccine types, oral polio vaccine (OPV), and IPV. OPV is cheaper, easier to administer, and provides mucosal and systemic immunity. These characteristics make it ideal for developing countries where resources are limited. However, it has been associated with a risk of reversion to virulence [81]. In contrast, IPV is a part of the immunization schedule employed by many developed countries and is a safer option but does not induce mucosal immunity. It has been suggested that the synergy of the two can be used in the campaign to eradicate polio. The main obstacles in this approach are the cost, the multiple doses (prime plus two boosters) and the cold chain storage (2–8°C) associated with IPV [82]. These obstacles often lead to incomplete immunizations i.e. not completing the full course required for protection. Recent studies have revealed a significant decrease in the number of children receiving the subsequent booster doses to those receiving the prime dose [83]. Evidence from a systematic review revealed that a single dose of IPV seroconverted on average 33%, 41%, and 47% of infants against serotypes 1, 2, and 3, respectively. Whereas two doses seroconverted 79%, 80%, and 90%, respectively [84]. Seroconversion reached 99–100% when three doses were administered according to immunization schedules [82]. This emphasizes the need for SAV technology with its potential to provide complete immunization and improve vaccine coverage rates. However, given the additional stability issues associated with IPV, no SAV formulations have been made until recently.

In a study by Tzeng S. et al., IPV was formulated in a pulsatile-release SAV and tested in animals versus two bolus injections administered with a 4-week gap. Both the absolute neutralizing response and the total IgG binding titers generated by the candidate formulations were measured. The absolute neutralizing response is regarded as the correlate of protection from IPV (level required: 1/4–1/8 dilution neutralization) [85]. They found that the newly developed system was non-inferior to the control of two bolus injections of IPV spaced one month apart based on animal studies that displayed strong and long-lasting immune responses generated by two SAV formulations.

Both SAV formulations contained PLGA but in combination with a different cationic polymer excipient. Eudragit E-containing microspheres generated a non-inferior absolute neutralizing response for IPV serotypes 1 and 2 during the 24 weeks of the experiment. On the other hand, they elicited an inferior response to IPV serotype 3 with a potentially shorter duration of immunity. Poly(α-l-lysine) with branched polyethylenimine (bPEI)-containing microspheres showed better results than the first formulation for IPV serotype 3 *in vitro* and appeared to have non-inferior IgG response to all three serotypes when compared to the control. It is important to note that both of the SAV formulations in this study initiated an antibody response earlier than the conventional vaccine. This observation requires further exploration because validating the capability of SAVs to initiate responses earlier than multi-dose vaccines can have a positive impact on their value, especially in cases where early responses are crucial [59].

The results from Tzeng et al. represent a considerable achievement in the development arena of SAV technologies for Polio. Yet, there are a number of questions for vaccine developers to consider, including whether immune response would last beyond 24 weeks and be non-inferior to the three-bolus IPV regimen of the routine immunization schedule. Although the *in vivo* performance of IPV depends predominantly on the neutralizing antibodies (according to the correlates of protection), investigating the immune response in greater detail may provide a better understanding of the total basis of immunity to polio and the opportunity to unveil additional benefits of SAV formulations.

### 3.2. Reactogenicity and granuloma formation in small animal studies

Vaccines are typically provided to healthy populations where there is a reasonable expectation for a high benefit-risk ratio. Reactogenicity is a term used to describe the adverse effects after vaccination and a high immunogenicity-reactogenicity ratio is desired. The development of adverse effects from vaccination varies between individuals and different types of vaccines, but it can be divided into two categories: local and systemic effects. Local effects may include swelling, pain, redness or localized hardening of soft tissues, whereas systemic effects may involve fever, fatigue, headache, and muscle pain. Reactogenicity arises from vaccines being recognized as potential pathogens by the body and inducing innate immune responses. Maintaining the balance between immunogenicity and reactogenicity is fundamental for a successful vaccine candidate [86]. Reactogenicity assessment is a long and dynamic process that spans through all the stages of vaccine development, from animal models to pharmacovigilance post-licensing [87].

Reactogenicity to SAVs is generally affected by two factors: the vaccine type itself and the excipients or adjuvants used in the formulation. For SAV formulations of IPV, the authors reported that there were no local or systemic adverse effects in their animal trials. According to Tzeng et al. this result was expected given the good safety profile of IPV and PLGA microspheres [59]. PLGA/PLA microspheres have a long history of biocompatibility, biodegradability, and safety that makes them one of the few FDA-approved drug delivery systems for parenteral administration [73].

Commercial HBV vaccines, being subunit vaccines, are inherently associated with low reactogenicity; however, the trade-off is that reduced reactogenicity is often coupled with reduced immunogenicity. To improve immunogenicity, this type of vaccine is often accompanied by adjuvants such as alum. The majority of SAV formulations reviewed above (in section 2) did not include alum and only one of those studies reported adverse reactions after SAV administration. Zheng et al. reported the appearance of lumps at the injection site in mouse models after administration of the SAV formulation but not for the active control. Interestingly, this study used the more reactogenic subcutaneous route together with a chitosan-alginate composite in addition to PLGA for their formulation. According to the authors, the lumps were not associated with skin rashes and disappeared within 6 weeks [70]. These may have formed due to possible deposits from SAV microspheres rather than granuloma formation.

Subcutaneous administration of vaccines has been associated with increased risk of granuloma formation [88]. Granulomas are pruritic nodules associated with local skin changes e.g. erythema, hypertrichosis, and discoloration [89]. Aluminium adjuvants have also been linked with increased rates of granuloma generation and a possible relation to chronic granulomatous inflammation, known as macrophagic myofasciitis [90,91]. It is important to note that there are new generations of adjuvants being developed many of them may be associated with increased reactogenicity at both local and systemic level [90]. In a study conducted by Boopathy et al., it was demonstrated that a modified release of vaccine enhanced the immune response through multiple mechanisms including facilitating germinal center B cell differentiation, promoting antibody class switching, and inducing the formation of long-lived plasma cells [46]. This may indicate that implementation of SAV for adjuvant delivery could allow a reduction in adjuvant dose and, therefore, the associated reactogenicity. This has the potential to enhance adherence to vaccines, ultimately resulting in increased vaccine coverage.

## 4. Translational aspects and commercialization

Several groups have published encouraging results on release properties of SAV formulations, followed by desirable immunogenicity and minimum reactogenicity in preclinical studies. However, these attempts have not yet progressed to human clinical trials. There can be various reasons for this lack of translation, including the cost of taking a product from bench to cGMP manufacturing and into Phase I studies. In the next sections, the key challenges that could influence commercial development of SAVs are highlighted.

### 4.1. Crossing the “valley of death” with new a formulation

The development of a new process that modifies an existing formulation may require establishment of a scalable manufacturing line, a robust and stable formulation and validated analytical assays, and additional clinical studies [92]. Addressing the technical challenges of a new process and navigating other obstacles encountered in the critical phase known as the ‘valley of death’ is crucial in determining whether a new product, process or a new formulation is commercially viable. Adapting an existing vaccine formulation to produce a SAV is considered to be a change in the formulation and manufacturing process. According to ICH guidelines, the manufacturer needs to evaluate the relevant quality attributes of the product and ensure there is no adverse impact on the safety and efficacy of the product [93]. Considerations for the clinical studies are assessed on a product-specific basis and the requirements may vary depending on the manufacturing process, the infectious disease to be prevented, the target population, the type of the vaccine and its mechanism of action. Changes in the formulation process, other than addition or removal of preservatives, may or may not give rise to a modified product that is viewed as a new candidate from a regulatory approval standpoint. Clinical bridging studies can be employed to directly compare the approved vaccine with the changed version in terms of efficacy and safety [94]. Manufacturing an SAV product to a cGMP standard using cGMP grade materials while simultaneously tackling inherent technical challenges for a Phase I trial can be economically and logistically demanding for an academic research group or a small and medium-sized enterprise (SME).

### 4.2. Scalability and feasibility to meet demand

Technology scalability was a major factor limiting the progression of a promising tetanus SAV formulation to clinical trials [57]. This approach employed spray drying, coacervation, and solvent evaporation to encapsulate the antigen in PLGA. The method was solvent based and required more space within a pilot scale clean-room GMP laboratory than the licensed vaccine formulation. It was calculated that changes in infrastructure could increase the price of the cheap tetanus vaccine by 100 times [10]. We believe that significant progress has been made in the field of spray drying/microencapsulation in general and in aseptic spray drying in the past 20 years, making this technique more mainstream, especially with emergence of Contract Development and Manufacturing Organizations (CDMOs) working in this area. While appreciating the complexity involved in vaccine manufacturing, it has been observed that manufacturing of vaccines/biologics has become less capital intensive and more agile through utilizing the efficiency of contract manufacturing, employing single-use technologies and continuous processing. The rate of new vaccine product development has been further enhanced with latest technologies, such as those observed during the coronavirus pandemic. As a step in the downstream process, spray-drying has emerged as a key player in pharmaceutical industry due to it being a single, continuous, and cost-effective process [95]. There are other niche and emerging techniques employed for formulation development, such as 3D printing and ALD. However, they suffer from slow production and increased operating costs [60,96]. Alternative technologies characterized for their upscaling capabilities such as microfluidics could be investigated further for SAV production to facilitate the bench to bed-side journey. Scaling-up can be achieved by parallelization i.e. several micromixers placed in parallel multiplying production rates. Microfluidics have also gained more prominence in the last few years mainly because of their capability to manufacture lipid carriers for vaccines [97].

In contrast to the traditional multiple dose vaccines, SAV formulations require additional excipients to control the release from the dosage form. Maintaining raw excipient quality can ensure production homogeneity, avoiding batch-to-batch variability. Sourcing those excipients in large quantities at GMP grade, sufficient to meet the demand of millions of doses per year for a routine immunization vaccine or billions of doses for a vaccine targeting a pandemic, can be exceptionally challenging [98]. Establishment of a robust excipient supply chain is critical for efficient manufacturing.

### 4.3. Cost analysis and market opportunities

Additional formulation steps employed in the manufacturing pipeline and the requirement for extra excipients mentioned above inevitably increase the initial manufacturing costs compared to some of the conventionally produced vaccines. A product-specific, cost-benefit analysis, encapsulating economic, environmental, societal impact, needs to be considered for each product. Reduced financial burden due to fewer clinical visits, decreased overhead costs of staff time and reduced logistical burden (i.e. storage, distribution) could offset the relatively higher cost of SAV formulations. According to the WHO, operational costs of vaccination campaigns contribute to approximately 90% of the total cost [26]. This will help in making a strong case for governments and organizations such as WHO, UNICEF, and GAVI to step in with covering the additional cost of manufacturing.

One approach to reduce manufacturing costs is to employ continuous methods for the microencapsulation of antigens such as microfluidics and spray-drying rather than batch methods. Yet, there will still be a cost/pricing gap to bridge between products manufactured using the above technologies and expected prices for traditionally manufactured vaccines. To put this into context, marketed products of long acting injectables which use PLGA for their controlled release technology such as Risperdal Consta® and Prostap SR® have UK drug tariff price ranges of £80–143 and £75–225 (equivalent to $91–162 and $85–256), respectively. Meanwhile, the average cost of vaccines production in developing countries is estimated at $2.18 per dose [99].

The real-world example of the pertussis vaccine (not SAV technology) illustrates that the cost is a key driver in vaccine candidate selection. Currently, there are two forms of the pertussis vaccine available: whole-cell (wP), and acellular (aP). According to the WHO, their protective efficacy is comparable, but the acellular vaccine has a better safety profile. However, the whole-cell vaccine remains the vaccine of choice in developing countries because of the lower cost [100]. An attractive market opportunity for SAV technologies is to focus on vaccines that are already expensive, (e.g. human papillomavirus and rabies vaccines) and target developed countries initially [61]. By employing this strategy, a proof of concept for the impact of SAVs could be provided, attracting attention from organizations such as GAVI that can potentially facilitate the introduction of SAVs to developing countries. Another opportunity is to formulate SAV technologies for the new generation vaccines (RNA, DNA) where there is currently a huge demand. For context, Pfizer’s Comirnaty® mRNA vaccine (COVID-19 vaccine) is currently considered the fastest-selling drug in pharma history with $36.9 Billion sales made in 2021. The current situation with COVID-19 vaccination provides evidence that SAV technology could have contributed to the faster control of the pandemic through maximizing vaccine coverage rates globally. However, retention of immunogenicity and stability of DNA/RNA vaccines in SAV technologies needs to be investigated.

### 4.4. Intellectual property and marketed products

Other than production costs, marketed long-acting injectable formulations are products that use patented technologies keeping their prices relatively high [8]. Medisorb^TM^ technology uses a water-based solvent extraction process to encapsulate a drug in PLGA microspheres. Upon administration, the microspheres absorb water and the polymer degrades in a controlled manner to release the drug over a period of days or months [101]. Marketed therapeutics utilizing this technology involve Comirnaty® and Risperdal Vivitrol®. Another technology which provided successful products (e.g. in Consta® Depot) is Eligard®. This technology introduces the drug in a PLGA/PLA solution containing a solvent that diffuses out when in contact with body fluids. As a result, the polymer precipitates out confining the drug in a solid implant with sustained and controlled release properties. A technology tailored to sensitive biomolecules and potentially suitable for SAV is Atrigel®. This technology involves pre-processing of the drug by spray freeze drying and addition to a PLGA solution followed by atomization. Low temperatures and nonaqueous conditions are maintained throughout the formulation process generating sustained-release microspheres [102]. However, ProLease® Depot which was a product of this technology has been discontinued for commercial reasons.

## 5. Conclusion

Over the past decades there have been considerable advances in the field of SAVs. Several groups have explored different microencapsulation methods and excipients generating useful information on how to tackle the key formulation challenges. The promising results from animal studies provide preliminary evidence that SAV formulations might be capable of producing strong and long-lasting immune responses in humans. The focus has now shifted toward translating SAV formulations in a marketed product. Utilizing scalable and continuous encapsulation methods, establishing a robust excipient supply chain and targeting suitable multiple dose vaccine candidates are approaches that may facilitate this journey.

Vaccines are becoming increasingly important for society, leading to more marketing and funding opportunities for SAVs. Taking advantage of these opportunities together with the utilization of expiring/expired patents for long-acting injectable formulations can enable researchers to make further steps toward SAV commercialization. If production development costs are reduced, SAVs will get the opportunity to reach the market and thrive commercially making a substantial contribution to global health outcomes.

## 6. Expert opinion

The translation of SAVs to commercial products has remained dormant for the last few decades since their initial conceptualization. The use of multivalent vaccines and compulsory immunization has been employed to increase coverage rates while SAVs have been neglected. The advances observed with the adoption of vaccination technologies during the recent pandemic can be a driving force to re-kindle the development space for SAVs. An additional, more efficient, technology to combat new infectious disease threats could make a game-changing contribution to the control of future outbreaks. The success of PLGA-based technologies in delivering several long acting injectables on the market despite the technological complexities should be replicated with SAVs. However, to achieve this goal, further considerations should be prioritized, including the cost implications of the above technologies on the final price of vaccines. One approach to minimize the cost per dose is to maximize the antigen loading capacity of the delivery vehicles/microspheres. Focusing on vaccines that are commercialized in the developed countries and that are at the higher price range may be an attractive market opportunity for SAV formulations at the early stage of development. However, availability of expensive vaccines for early-stage R&D could be challenging. Generation of proof-of-concept data with model protein followed by generation of reproducible data with relevant less expensive vaccine prior to embarking on expensive vaccines presents as a more sensible approach.

It is vital to achieve a controlled release profile (pulsatile or sustained) that is capable of generating superior immune response to a multiple dosing regimen of conventional vaccines. Achieving better control over release and higher encapsulation efficiency has become more feasible due to the emergence of technologies such as microfluidics and particle engineering techniques such as spray drying. An additional critical challenge that should be prioritized is the stability of antigens within the developed microspheres, which should help reduce the operational, logistical, and especially manufacturing costs, which are relatively high.

Importance of single administration technology is increasingly getting more attraction and the advancement of the technology in the field of vaccines is expected to revive in the next coming years. SAVs provide great additional values to existing vaccine products by generating modified (pulsatile or sustained) release potentially outperforming traditional strategies. The SAV formulation needs further development and assessment with more advanced vaccine candidates such as nucleic acids, potentially increasing stability, immunogenicity, and efficacy.

The technology as it stands has come a long way from its inception in 1980s. There are still a number of challenges some of which have been elucidated in this document. Depending on the complexities of the vaccines and their tolerance to different process employed for the manufacture of formulations capable of modified release, the technology is not expected to be applicable for all vaccines. Adopting and further developing the technology utilizing more robust vaccines such as toxoid and subunit vaccines will certainly pave path to more complex nucleic acid vaccines such as RNA formulated in liquid nano particles and subunit vaccines combined with more robust adjuvants tailored to produce disease specific desirable immunological response.

## Article highlights

- Multiple dose COVID-19 vaccines are potential candidates of single administration vaccine technology.
- Modifying release to generate a sustained or pulsatile release profile to minimize burst release is one of the major formulation challenges.
- Maintaining stability of sensitive antigens during different stages of preparation, storage, and *in vivo* environment is a key challenge associated with SAV.
- Promising SAV attempts have not progressed to human clinical trials yet probably because of cost implications on the final product and the translational cost of taking a product from bench to cGMP manufacturing and into Phase I studies.
- The renewed global focus on vaccinations since the COVID-19 pandemic may provide an impetus for SAV commercialization for pandemic preparedness in the future.
